# Phylogenetic and Temporal Dynamics of Human Immunodeficiency Virus Type 1 CRF01_AE in China

**DOI:** 10.1371/journal.pone.0054238

**Published:** 2013-01-24

**Authors:** Jingrong Ye, Ruolei Xin, Shuangqing Yu, Lishi Bai, Weishi Wang, Tingchen Wu, Xueli Su, Hongyan Lu, Xinghuo Pang, Hong Yan, Xia Feng, Xiong He, Yi Zeng

**Affiliations:** 1 Beijing Center for Disease Prevention and Control, Beijing, China; 2 College of Life Science and Bio-engineering, Beijing University of Technology, Beijing, China; 3 State Key Laboratory for Infectious Diseases Prevention and Control, National Institute for Viral Disease Control and Prevention, China Center for Disease Prevention and Control, Beijing, China; 4 Program of Pharmacology and Toxicology, University of Toronto, Toronto, Canada; National HIV and Retrovirology Laboratories, Canada

## Abstract

To explore the epidemic history of HIV-1 CRF01_AE in China, 408 fragments of gag gene sequences of CRF01_AE sampled in 2002–2010 were determined from different geographical regions and risk populations in China. Phylogenetic analysis indicates that the CRF01_AE sequences can be grouped into four clusters, suggesting that at least four genetically independent CRF01_AE descendants are circulating in China, of which two were closely related to the isolates from Thailand and Vietnam. Cluster 1 has the most extensive distribution in China. In North China, cluster 1 and cluster 4 were mainly transmitted through homosexuality.The real substance of the recent HIV-1 epidemic in men who have sex with men(MSM) of North China is a rapid spread of CRF01_AE, or rather two distinctive natives CRF01_AE.The time of the most recent common ancestor (tMRCA) of four CRF01_AE clusters ranged from the years 1990.9 to 2003.8 in different regions of China. This is the first phylogenetic and temporal dynamics study of HIV-1 CRF01_AE in China.

## Introduction

China is the world's most populous country, where the human immunodeficiency virus type 1 (HIV-1) epidemic is still increasing. At the end of 2011, the estimated number of people living with HIV in China was 780,000. Women accounted for 28.6% of these cases. Prevalence among the population as a whole was 0.058%; Of the total number of people living with HIV, 154,000 were cases of Acquired Immune Deficiency Syndrome(AIDS). It is estimated that there were 48,000 new cases of HIV in 2011, and that the prevalence among the population as a whole was 0.057%. HIV/AIDS is also the leading cause of death from infectious diseases in China, accounting for an estimated 28,000 deaths in 2011. Sexual transmission continues to be the primary mode of transmission, and homosexual transmission is also increasing rapidly. Of the 780,000 people estimated to be living with HIV in 2011, the percentage of infected cases through sexual transmission was 63.9% in which 46.5% were infected through heterosexual transmission and 17.4% through homosexual transmission. The percentage of infected cases through intravenous drug user (IDU) was 28.4%. Among the 48,000 new cases estimated for 2011, heterosexual transmission accounted for 52.2%, homosexual transmission 29.4%, and IDU accounted for 18.0% of cases [1].

HIV-1 CRF01_AE is the earliest circulating recombinant form (CRF) among those identified to date. They represent a putative subtype of A/E recombinant that was originated from Central Africa but spreading epidemically in Asia. [2]. CRF01_AE plays important role in regional epidemics and is responsible for 5% of cases in the world. The majority (83%) of CRF01_AE was found in South and Southeast Asia, whereas 9% in East Asia. In Cambodia, Thailand, and Vietnam, CRF01_AE is responsible for more than 95% of the infections [3]. CRF01_AE was first identified from the women who had returned to Yunnan province, China from Thailand after involving in commercial sex works in late 1994 [4]. Later, CRF01_AE strains were found in injecting drug users (IDUs) from Guangxi province in 1996 [5].

In the recent years, HIV-1 CRF01_AE spreads rapidly since the early 2000s in China. The recent molecular epidemiology survey in Beijing identified HIV-1 CRF01_AE (40.4%) as the most dominant strains. In men who have sex with men (MSM), CRF01_AE was accounted for 56.8% of all infections (unpublished data).The factors associated with the rapid increase of CRF01_AE in China are not completely known, but in certain regions where it has been introduced, CRF01_AE has overtaken other HIV-1 strains introduced earlier. The rapid spread of CRF01_AE in many regions of China including Beijing, Guangxi, Guangdong, Jiangxi, Hunan and Hainan has drawn particular attention [6]–[9]. Recent studies suggested that Hong Kong CRF01_AE epidemic likely derived from multiple origins with 3 separate transmission clusters identified [10]. In Guangxi region also there were multiple introductions of CRF01_AE strains and a peculiar CRF01_AE monophyletic lineage distinct from other CRF01_AE viruses was identified [11]. However, the genetic characteristics of CRF01_AE in other parts of China remained mysterious.

By combining phylogenetic analyses and a Bayesian coalescent-based approach, the phylogenetic relationships of CRF01_AE isolates from China were studied in order to provide more understanding on the epidemic growth model of HIV-1 CRF01_AE virus.

## Results

### 1. Study subjects

A total of 408 CRF01_AE gag gene (836–1486 nt, HXB2) sequences isolated from 27 provinces of China during the year 2002–2010 were used in this study. 186 cases were newly characterized from ongoing molecular epidemiology studies mainly in Beijing, the capital city of China, and 222 cases were obtained from the Los Alamos HIV sequence database (www.hiv.lanl.gov) from previously published reports. The clinical and demographic characteristics of the 186 HIV-1 CRF01_AE Infectors reported by our laboratory were as follows: mean age was 32.0 (2–69) years and 91.9% (171 of 186) were male. The predominant route of transmission was MSM in (64.0%,119 of 186) of the subjects, while the remaining were heterosexual(25.8%, 48 of 186),IDUs (3.2%, 6 of 186), Mother to child transmission(0.5%,1 of 186), and blood transmission (0.5%,1 of 186). Median CD4 count was 328 cells/mm3 (table 1). The province of origin and sampling year of the 222 CRF01_AE sequence are listed in table 2. Geographically, the 408 CRF01_AE gag gene sequences originated from different regions of China are as follows: the North China (Beijing n = 67, Hebei n = 33,Shanxi n = 1, Inner Mongolia n = 2); Northeast China (Liaoning n = 60, Jilin n = 5, Heilongjiang n = 5); East China (Shanghai n = 1, Jiangsu n = 5, Zhejiang n = 5, Anhui n = 2, Fujian n = 14, Jiangxi n = 2, Shandong n = 7); South Central China (Henan n = 9, Hubei n = 2, Hunan n = 2, Guangdong n = 1, Hainan n = 1 Guangxi n = 144); Southwest China (Chongqing n = 2, Sichuan n = 14,Yunnan n = 4); Northwest China (Shannxi n = 3, Gansu n = 5, Ningxia n = 2, Xinjiang n = 1);Unknown(n = 7) (Table 3).

**Table 1:** Demographic and clinical information for the 186 HIV-1 CRF01_AE Infectors reported by our laboratory.

	Overall(186)
Age [median (range)], yrs	32(2–69)
gender n (%)	
male	171(91.9)
famale	15(8.1)
Risk factors n (%)	
Hetero	48(25.8)
MSM	119(64.0)
IDUs	6(3.2)
Mother to child	1(0.5)
Blood	1(0.5)
Unknown	11(5.9)
Median CD4 count (cells/mm^3^)	328
Range of sampling date	2006–2010
Province	
Beijing	67(36.0)
Hebei	24(12.9)
Henan	9(4.8)
Sichuan	14(7.5)
Other 23 Province	72(38.7)

**Table 2:** Sampling information for the 222 HIV-1 CRF01_AE sequences obtained from the Los Alamos HIV sequence database.

	Overall(222)
Province	
Fujian	12(5.4)
Guangxi	144(64.9)
Hebei	9(4.1)
Jiangsu	1(0.5)
Liaoning	56(25.2)
Range of sampling date	2002–2010

**Table 3:** Distribution of CRF01_AE isolates (cluster 1 to 4) in different regions of China.

Region	overall	cluster 1	cluster 2	cluster 3	cluster 4	other
North China	103	75	5	3	18	2
Beijing	67	46	4	2	13	2
Hebei	33	26	1	1	5	
Shanxi and Inner Mongolia	3	3				
Northeast China	70	12	2	4	48	4
Liaoning	60	5	2	4	46	3
Jilin and Heilongjiang	10	7			2	1
East China	36	20	1	5	3	7
Fujian	14	1	1	4	1	7
Shandong	7	7				
Zhejiang,Anhui,Jiangxi,Shanghai,Jiangsu	15	12		1	2	
South Central China	161	14	68	70	2	7
Guangxi	146	7	66	67		6
Henan	9	2	1	3	2	1
Hubei,Hunan,Guangdong,Hainan	6	5	1			
Southwest China	20	12	2	1	3	2
Sichuan	14	8	2	1	2	1
Qongqing and Yunnan	6	4			1	1
Northwest China	11	7			3	1
Shaanxi,Gansu,Ningxia,Xinjiang	11	7			3	1
Unknown	7	6			1	
Total	408	146	78	87	78	19

### 2. Molecular Epidemiology of the HIV-1 CRF01_AE Infection in China

**Figure 1:**
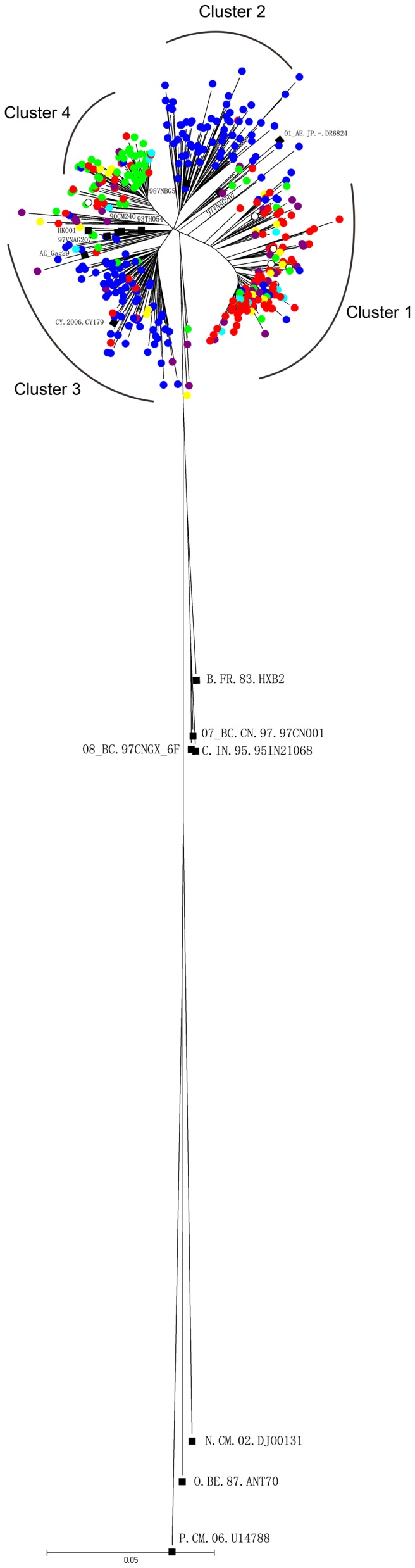
Phylogenetic tree analysis of Chinese HIV-1 CRF01_AE gag gene sequences. The phylogenetic tree was constructed using neighbor-joining methods (Mega 4.0) for the gag region. Samples of North China,Northeast China,South Central China,East China,Southwest China and Northwest China are shown by red, green, blue, purple,yellow and cyan solid circles in the tree. The empty circle indicate unknown. The black solid squares indicate reference sequences from the Los Alamos HIV sequence database.

A phylogenetic tree was reconstructed using NJ method with 418 gag sequences, consisting of 408 China sequences and 10 CRF01_AE reference sequences isolated in Thailand, Vietnam, Cyprus, Japan and Hong Kong. The tree demonstrated 19 out of 408 China sequences (4.7%) which were scattered among each other while the other 389 sequences were segregated into four major distinct clusters (indicated as cluster 1 to 4) with bootstrap value above 70% and more than 20 members (Figure 1). Among the 4 clusters, cluster 1 had 146 members that isolated from North China(75), Northeast China(12), East China(20), South Central China(14), Southwest China(12) and Northwest China(7). the epidemic information of 127 individuals which was available, the median age range was 31.6 (20–62) years,124 were males and 3 were females. Heterosexual (18.1%,23 of 127) and MSM (71.7%, 91 of 127) accounted for the major transmission routes for these isolates. For cluster 2, 78 sequences were mainly isolated from South Central China (84.6%,66 of 78) and 12 people from other province of China. This cluster also contains one sequence (98VNBG5) from Vietnam. Cluster 3 included 87 sequences mainly from Guangxi(77.0%,67 of 87) and were found to cluster with 7 reference sequences isolated from HIV-1-infector in Thailand (90CM240,93TH054,93TH051, TH.2006.AE_Gag29),Vietnam(97VNHCM319),Cyprus (CY.2006.CY179) and Hong Kong(HK.2004.HK001). Cluster 4 included 78 sequences mainly from Northeast China (61.5%,48 of 78) and North China(23.1%,18 of 78), the 33 infectors of which the epidemic information was available, were all male with the median age of 31.4(20–61) years, 23 belonged to the MSM risk group, whereas 9 were infected through heterosexual contacts. Phylogenetic analyses of the viral gag genes showed that clusters 2 and 3 were closely related to the strains identified in Thailand and Vietnam, suggesting that the two clusters have close genetic relationships with these strains. The CRF01_AE sequences in cluster 1 and cluster 4 were different from those belonging to Thailand and Vietnam. These two clusters were pure Chinese CRF01_AE clusters which do not contain any foreign strains. As far as the transmission routes were concerned in North China, the cluster 1 and cluster 4 were mainly transmitted by homosexual, cluster 2 by heterosexual, and cluster 3 by combined methods including heterosexual, homosexual and IDUs (table 4).The Maximum Clade Credibility with time scale (MCC) tree of six region of China were included in this study as shown in figure S1, S2, S3, S4, S5, S6.

**Table 4:** Demographic and clinical information for patients infected with CRF01_AE virus in North China.

	Overall(103)	cluster 1(75)	cluster 2(5)	cluster 3(4)	cluster 4(18)
Age [median (range)], yrs	33.7(20–69)	33(20–62)	31.4(25–45)	46(27–69)	34.4(20–61)
gender n (%)					
Male	98(95.1)	74(98.7)	4(80.0)	2(50.0)	18(100.0)
Female	5(4.9)	1(1.3)	1(20.0)	2(50.0)	
Risk factors n (%)					
Heterosexual	23(22.3)	11(14.7)	5(100.0)		7(38.9)
MSM	73(70.9)	60(80.0)		2(50.0)	11(61.1)
IDUs	2(1.9)	1(1.3)			
Blood	1(1.0)			1(25.0)	
Unkown	4(3.9)	3(4.0)		1(25.0)	
Median CD4 count (cells/mm^3^)	316	337	168	181	386
Range of sampling date	2006–2010	2006–2010	2006–2010	2007–2009	2006–2010

### 3. Regional distribution of HIV-1 CRF01_AE in China: Analysis by Region

Marked differences were found between the six region in China. Table 3 gives the details of the distribution of HIV-1 CRF01_AE within each region. . For the North China, 72.8%(75 of 103) of CRF01_AE infections were caused by cluster 1, with smaller proportions of infections caused by cluster 4 (17.5%,18 of 103), cluster 2(4.9%,5 of 103) and cluster 3(2.9%,3 of 103) strains(table 4). While, in Northeast China, the dominant CRF01_AE strains were cluster 4(68.6%,48 of 70) and cluster 1 accounts for 17.1%(12 of 70). In South Central China, the most prevalent CRF01_AE strains were cluster 3(43.5%,70/161) and cluster 2(42.2%,68/161).In East China, Southwest China and Northwest China, the epidemic of CRF01_AE is dominated by cluster 1, which was found to be responsible for 55.6%(20 of 36), 60.0%(12 of 20) and 63.6%(7 of 11) of all infections, respectively.

### 4. Date of Origin of the Four HIV-1 CRF01_AE Strains in China

The four taxons identified were used for the tMRCA calculations. The mean estimated evolutionary rate for four clusters in different regions ranged from 2.31×10^−3^ to 3.34×10^−3^ substitutions site^−1^ year^−1^ under the relaxed exponential clock model (table 5). The Bayes factor analysis showed that the relaxed exponential clock model was strongly supported by other model for this dataset. The estimated dates of introduction of cluster 1 into different regions were as follows: 1990.9 into East China, 1992.7 into North China and Southwest China, 1998.8 into South Central China, 2003.8 into Northeast China. Cluster 2 into South Central China was dated back to 1998.0. Cluster 3 has an estimated tMRCA around 1997.9 in South Central China. The median estimation of the tMRCA for the cluster 4 in North China and Northeast China were 1998.8 and 1995.3, respectively (table 5). This is in agreement with previous epidemiological investigations on the first report of CRF01_AE in the middle of 1990s in China.

**Table 5:** Estimated Substitution Rates and Dates for Transmission Clusters.

	Bayesian Coalescent*	
Region	Rate of evolution	tMRCA
North China		
Cluster 1	2.31×10^−3^	1992.7(1989.5–1995.9)
Cluster 4	3.17×10^−3^	1998.8(1998.1–1999.5)
Northeast China		
Cluster 1	2.46×10^−3^	2003.8(2003.3–2004.3)
Cluster 4	3.29×10^−3^	1995.3(1995.1–1995.5)
East China		
Cluster 1	2.89×10^−3^	1990.9(1987.9–1993.9)
South Central China		
Cluster 1	2.56×10^−3^	1998.8(1997.2–1999.4)
Cluster 2	3.34×10^−3^	1998.0(1996.0–2000.0)
Cluster 3	2.86×10^−3^	1997.9(1996.12–1998.7)
Southwest China		
Cluster 1	2.77×10^−3^	1992.3(1990.3–1994.3)

* Estimates of the mean evolutionary rate (μ, substitutions.site^−1^.year^−1^) and the median time of the most recent common ancestor (tMRCA) for the different clusters (95% high posterior density in parentheses).

### 5. Epidemic history of of HIV-1 CRF01_AE cluster 4 in Northeast China

**Figure 2:**
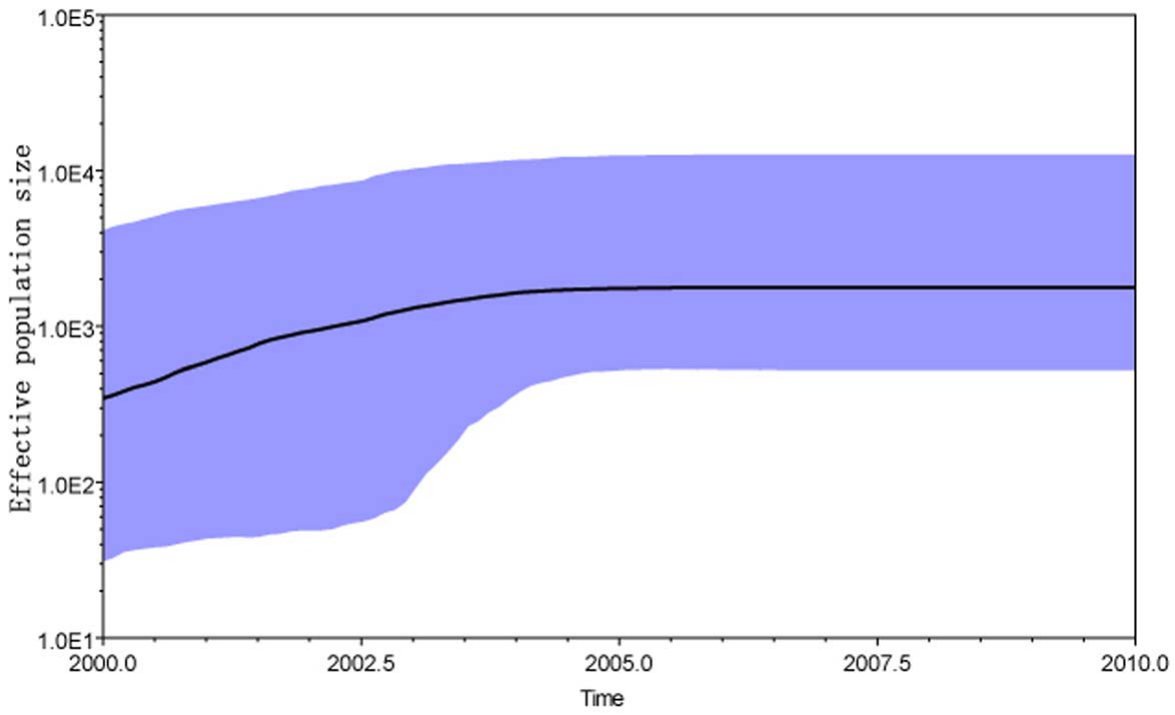
Baysian skyline plot was estimated to reconstruct the demographic history of CRF01_AE cluster 4 in Northeast China. The x axis is the time in units of years, and the y axis is equal to the effective population size. The thick solid line is the mean estimates, the 95% HPD credible region is showed by blue areas.

Bayesian skyline plot (BSP) analysis was also used to infer the estimation of the effective population size at the time of CRF01_AE epidemics in Northeast China, for the as sampling time span was ranged from 2002–2010.The demographic history from the gag BSP identified three epidemic growth phases (figure 2), an initial slow growth phase until the year 2002, followed by an exponential growth phase till 2005, followed by a stationary phase, approaching the present time.

## Discussion

To the best of our knowledge, this is the first nationwide phylodynamic study depicting the spatiotemporal dynamics of HIV-1 CRF01_AE in China and to trace the tMRCA of the CRF01_AE strains. The analyses using phylogenetic reconstruction and coalescence inference indicate that the spread of CRF01_AE in China involved at least 4 viral lineages. Cluster 1 strains play a visible role in the CRF01_AE epidemic in China, and cause a significant proportion of infections in Southwest China (60%), East China (55.6%), Northwest China (63.6%), and especially in North China (72.8%). Both cluster 2 and cluster 3 are found mainly in South Central China and are responsible for about 42.2% and 43.5% of infections, respectively.Cluster 4 was prevalent in Northeast China (68.6%) and North China(17.5%).

The tMRCA of cluster 1 ranged from 1990.9 to 2003.8 in different regions of China. The CRF01_AE cluster 2 lineage likely entered the South Central China around 1998.0. The cluster 3 CRF01_AE epidemic in South Central China was probably established around 1997.6. Cluster 4 was dated back to 1995.3 and probably introduced later into neighboring North China region around 1998.8. Cluster 4 was a latest cluster that was mainly confined to Northeast China and North China. But it remained unclear whether the isolate spread to other regions.

Previous studies have defined the tMRCA of the two clades of CRF01_AE strains from Viatnam in 1989.8 and 1997.5, respectively [12], [13].A recent report regarding the tMRCA of HIV-1 CRF01_AE in Hong Kong dates back to late 1980s and late 1990s [10]. In this study, the time of origination of the China HIV-1 CRF01_AE was defined as from 1990.9 to 2003.8.This evidence suggested that CRF01_AE strain might begin to circulate in mainland China at nearly the same time as Hong Kong. Our results also conform the long time assumption that CRF01_AE in China was originated from Southeast Asia. Zeng H et al. identified three HIV-1 CRF01_AE variants in Guangxi province in 293 CRF01_AE samples recruited between 2009 and 2010, of which one was novel strain distinct from other CRF01_AE viruses [11]. Indeed, nearly all of these strains in Guangxi were included in the current study and the strains mostly belong to cluster 2 and cluster 3.

Nonetheless, a comment should be made about the limitation of the data acquisition in this study. Although the entire China CRF01_AE gag sequences from NCBI GenBank were included when this study was initiated, the number of the sequences was relatively small, especially for East China, Southwest China, and Northwest China, until more archival specimens from these regions were retrieved and thoroughly analyzed, the complex dissemination of CRF01_AE lineages were interpreted with caution.

## Materials and Methods

### 1. Ethics Statement

This study was approved by the Committee on Human Research at the Beijing Center for Disease Prevention and Control (Beijing CDC) and written inform consents were obtained from all participants.

### 2. Study Population

In total, 408 CRF01_AE gag gene sequences sampled within the China during 2002–2010 were obtained (GenBank accession numbers are JF759957–JF760203), including 186 from the molecular epidemiology research conducted in Beijing and 222 for which province of origin and sampling year were known, from Los Alamos HIV sequence database. The patient epidemiological information including age, gender, ethnicity, place of birth, route of infection, and CD4 cell count were also collected.

### 3. Sequence Alignment and Phylogenetic Tree Analysis

The 408 local CRF01_AE gag sequences were aligned with 10 HIV-1 CRF01_AE reference sequences isolated from Thailand, Vietnam, Cyprus, Japan and Hong Kong using Vector NTI 8.0 software (Invitrogen, USA). Multiple alignments were performed automatically by BioEdit with minor manual adjustments. A phylogenetic tree based on gag sequence was constructed with Kimura 2- Parameter model and Neighbor-Joining (NJ) method, using MEGA 4. The bootstrap test was performed with 1,000 replications [14]. Reference sequences of different subtypes (B,C) and CRFs(CRF07_BC, CRF08_BC) strains were downloaded from HIV-1 Los Alamos Database as outgroup sequences.

### 4. Estimation of the Transmission History of CRF01_AE in China

The evolution rate, time of most recently common ancestor (tMRCA), and demographic history of the CRF01_AE strains circulating in mainland China were inferred using Bayesian Markov chain Monte Carlo (MCMC) method in BEAST version 1.4.8.The Relaxed Clock: Uncorrelated Exponential model was tested in combination with four different coalescent tree priors (‘Constant Size’; ‘Exponential Growth’, ‘Logistic Growth’ and ‘Bayesian Skyline’) under an HKY nucleotide substitution model with heterogeneity among sites modeled with a gamma distribution and invariant sites. For each model the MCMC chain was run for 20,000,000 steps and sampled every 2,000 steps. The first 2,000,000 steps of each run were discarded as burn-in. The resulting log-files were analyzed in Tracer v.1.6.2 and the Bayes Factor was calculated to compare molecular clock models, using marginal likelihood as implemented in Tracer v.1.5. The Maximum Clade Credibility with time scale (MCC) tree was obtained by TreeAnnotator v1.6.1 with a burn-in of the first hundred trees [15]–[17].

## Supporting Information

Figure S1 **Bayesian phylogenetic tree of HIV-1 CRF01_AE gag sequences isolated from North China.**
(PDF)

Figure S2 **Bayesian phylogenetic tree of HIV-1 CRF01_AE gag sequences isolated from Northeast China.**
(PDF)

Figure S3 **Bayesian phylogenetic tree of HIV-1 CRF01_AE gag sequences isolated from East China.**
(PDF)

Figure S4 **Bayesian phylogenetic tree of HIV-1 CRF01_AE gag sequences isolated from South Central China.**
(PDF)

Figure S5 **Bayesian phylogenetic tree of HIV-1 CRF01_AE gag sequences isolated from Southwest China.**
(PDF)

Figure S6 **Bayesian phylogenetic tree of HIV-1 CRF01_AE gag sequences isolated from Northwest China.** Colors indicate the geographic location of sampling as follow: cluster 1 in red, cluster 2 in green, cluster 3 in purple and cluster 4 in blue.
(PDF)
