# Supplementary figures and images for: Phylogenetic and Temporal Dynamics of Human Immunodeficiency Virus Type 1 CRF01_AE in China
Source: PLoS One. 2013 Jan 24;8(1):e54238. doi: 10.1371/journal.pone.0054238 (PMC3554705; doi:10.1371/journal.pone.0054238)

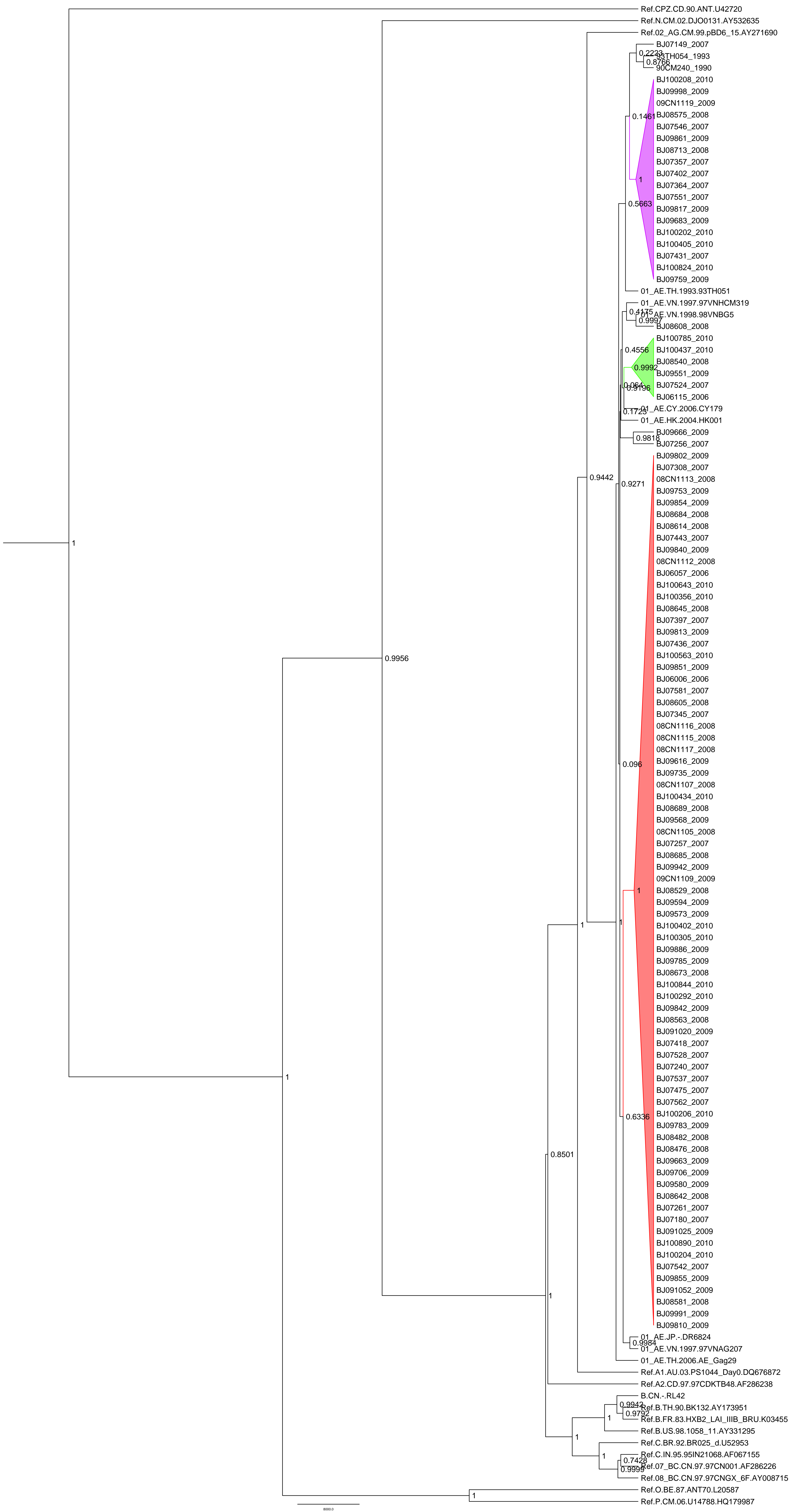

Supplement: Figure S1 — Bayesian phylogenetic tree of HIV-1 CRF01_AE gag sequences isolated from North China. (PDF) [file pone.0054238.s001.pdf]

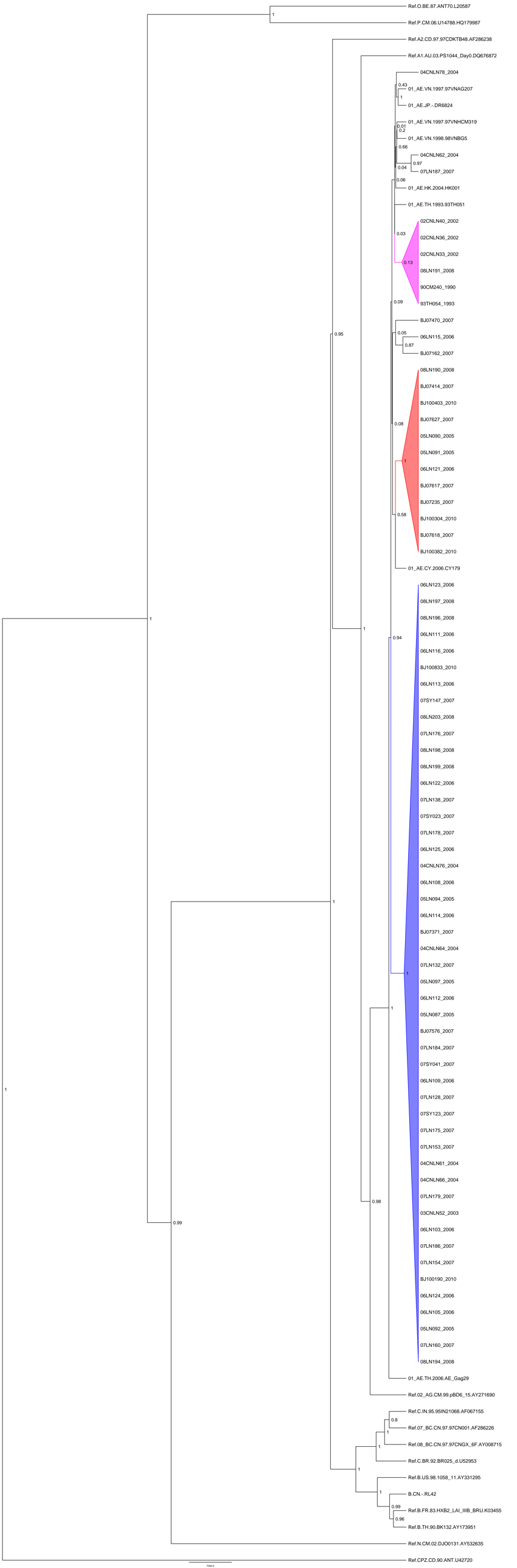

Supplement: Figure S2 — Bayesian phylogenetic tree of HIV-1 CRF01_AE gag sequences isolated from Northeast China. (PDF) [file pone.0054238.s002.pdf]

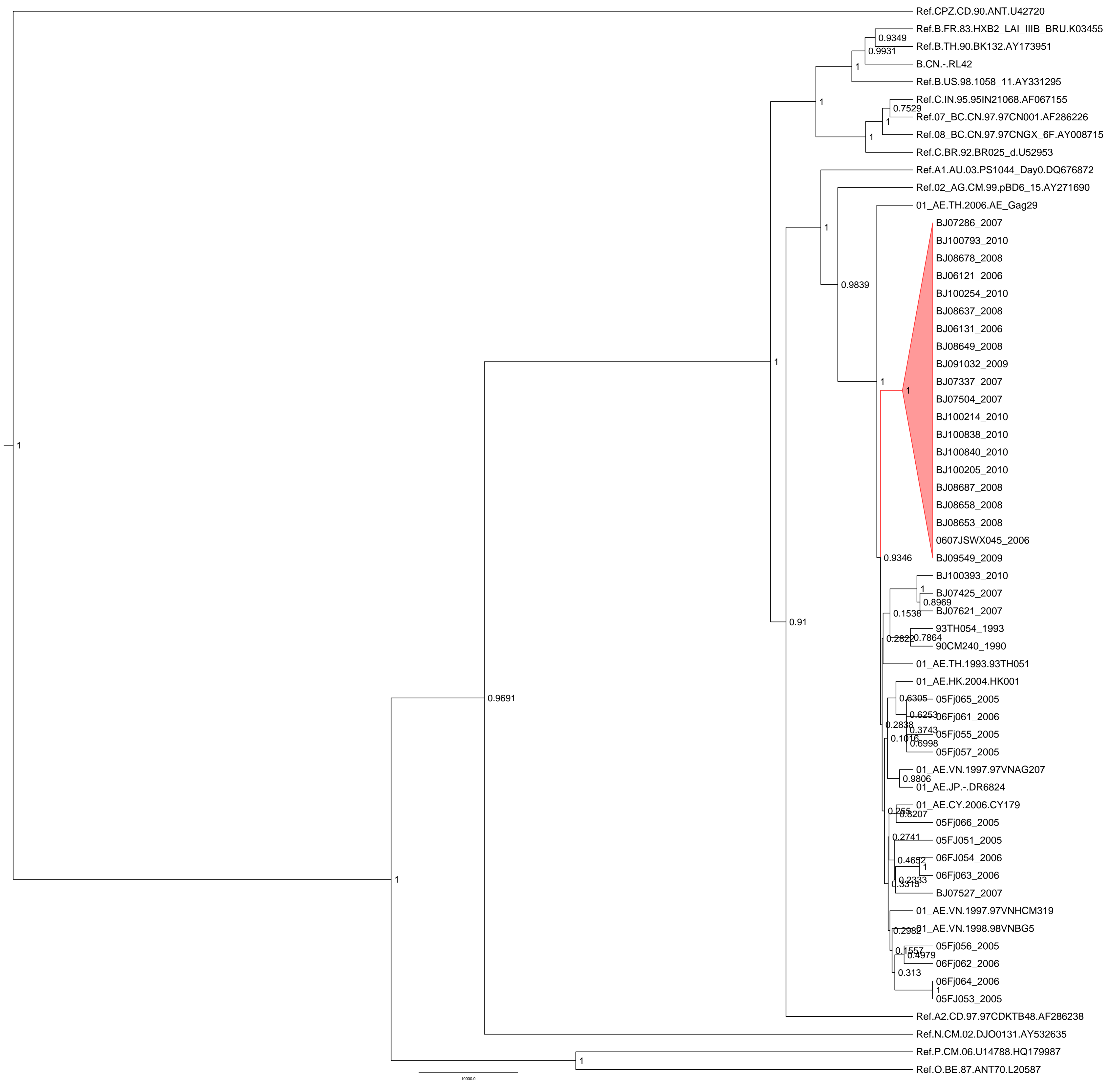

Supplement: Figure S3 — Bayesian phylogenetic tree of HIV-1 CRF01_AE gag sequences isolated from East China. (PDF) [file pone.0054238.s003.pdf]

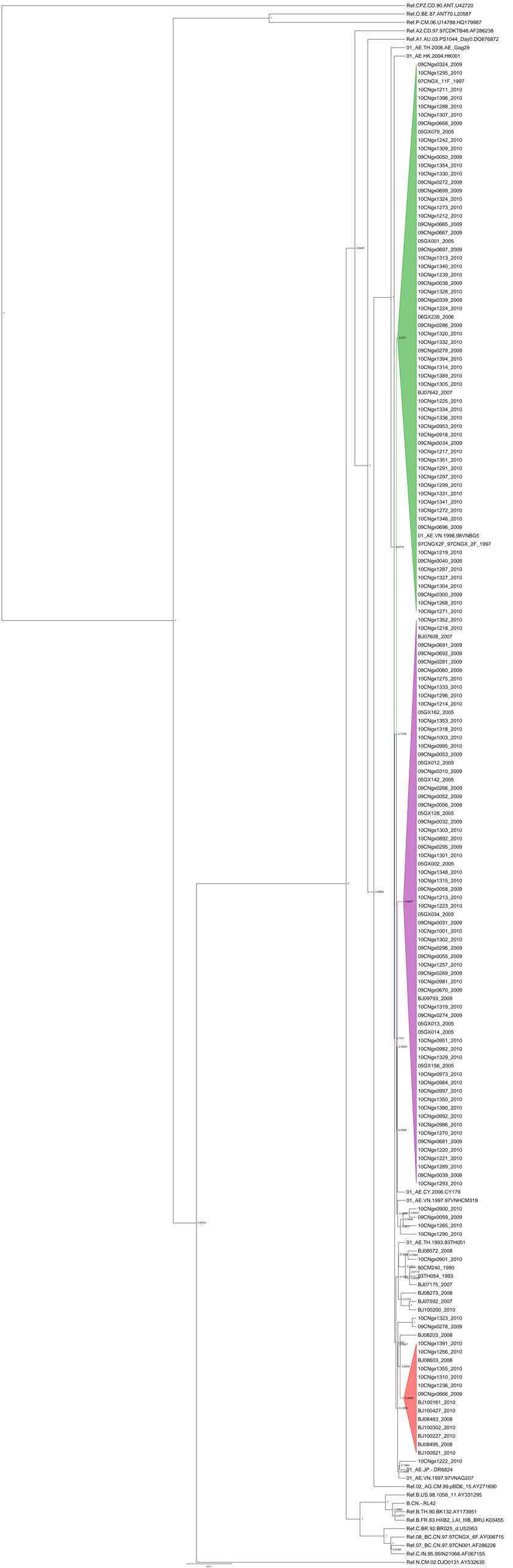

Supplement: Figure S4 — Bayesian phylogenetic tree of HIV-1 CRF01_AE gag sequences isolated from South Central China. (PDF) [file pone.0054238.s004.pdf]

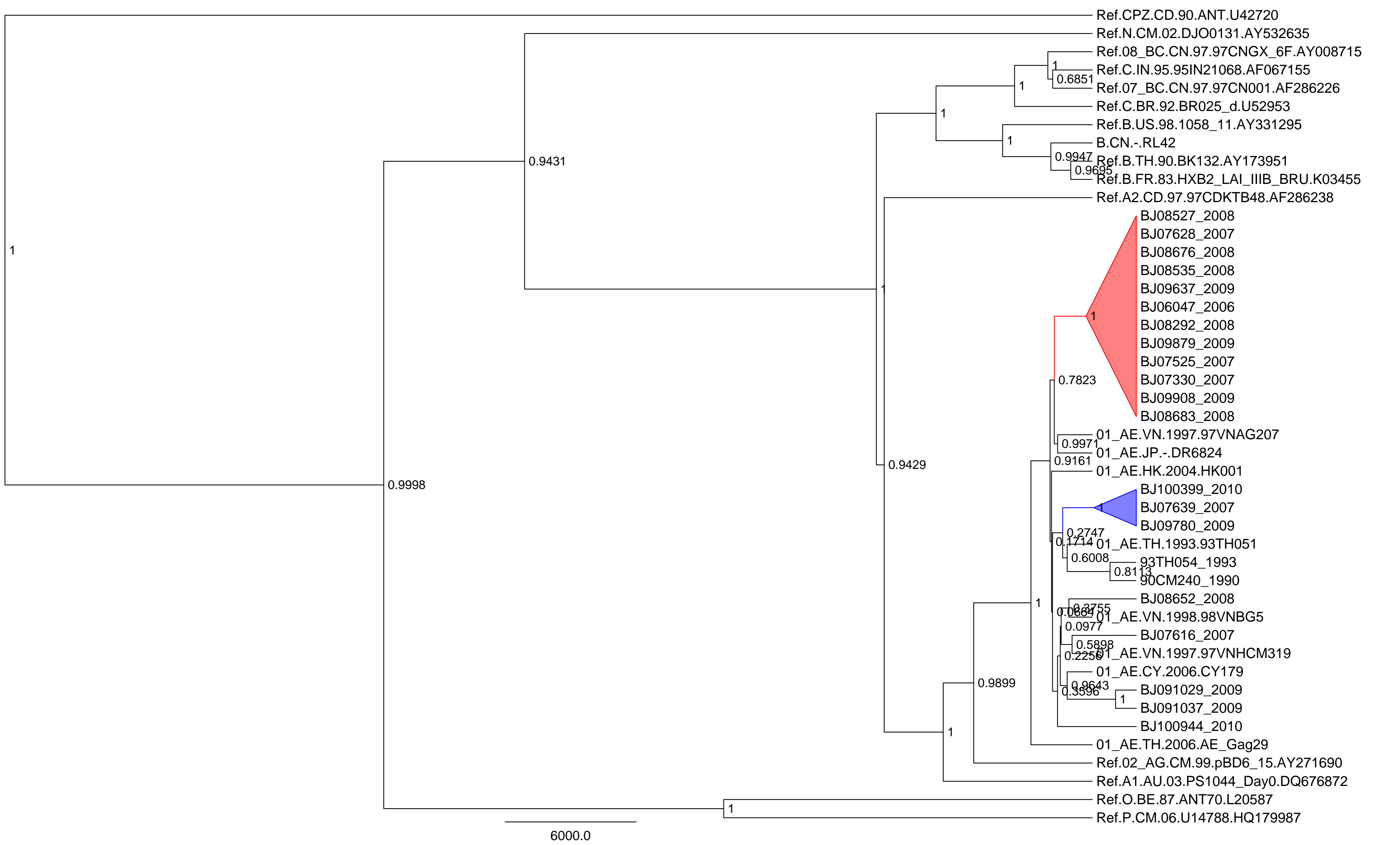

Supplement: Figure S5 — Bayesian phylogenetic tree of HIV-1 CRF01_AE gag sequences isolated from Southwest China. (PDF) [file pone.0054238.s005.pdf]

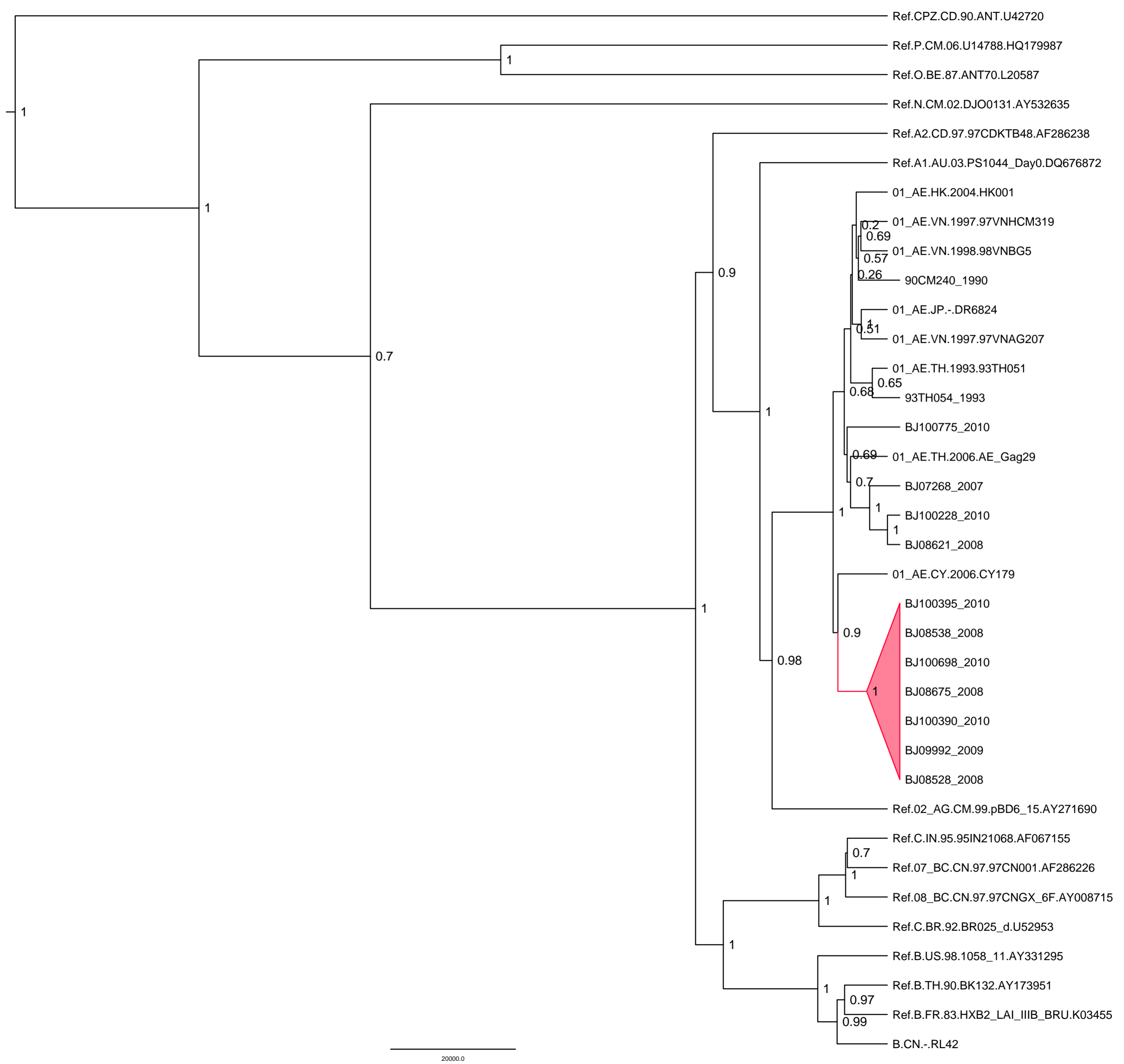

Supplement: Figure S6 — Bayesian phylogenetic tree of HIV-1 CRF01_AE gag sequences isolated from Northwest China. Colors indicate the geographic location of sampling as follow: cluster 1 in red, cluster 2 in green, cluster 3 in purple and cluster 4 in blue. (PDF) [file pone.0054238.s006.pdf]
